# Cross-Sectional Serological Survey of Human Fascioliasis in Haiti

**DOI:** 10.1155/2012/751951

**Published:** 2011-08-25

**Authors:** P. Agnamey, E. Fortes-Lopes, C. P. Raccurt, J. Boncy, A. Totet

**Affiliations:** ^1^CHU Amiens, Hôpital Sud, Service de Parasitologie et Mycologie Médicales, Avenue René Laënnec, Salouël, 80054 Amiens, France; ^2^Laboratoire National de Santé Publique, Delmas 33, Port-au-Prince, Haiti

## Abstract

*Fasciola hepatica*, the aetiological agent of fascioliasis in the Caribbean region, occurs throughout the major islands of the Greater Antilles and in localised zones on two islands (Martinique and Saint Lucia) of the Lesser Antilles. However, apart from Puerto Rico, information regarding human fascioliasis in islands of the Caribbean is out of date or unavailable, or even nonexistent as in Haiti. The authors conducted a retrospective, cross-sectional serological survey in Port-au-Prince using a Western blotting test (LDBIO Diagnostics) on human fascioliasis in Haiti. A total of 216 serum samples obtained from apparently healthy adults were tested. The frequency of antibodies in serum samples of the study population was 6.5% (14/216). The immunodominant bands recognised in Western blots were 27-28 kDa (100%), 42 kDa (64%), 60 kDa, and 8-9 kDa (28%). This is the first survey to reveal a relatively low proportion of asymptomatic *F. hepatica*-infected humans in Haiti.

## 1. Introduction

Fascioliasis, a snail-borne parasitic zoonosis, has been recognised for a long time because of its major veterinary impact. It is caused by two trematode species of the *Fasciola* genus: *F. hepatica*, present in Europe, Africa, Asia, the Americas, and Oceania and *F. gigantica*, mainly distributed in Africa and Asia. Fascioliasis is currently the vector-borne disease with the widest known latitudinal, longitudinal, and altitudinal distribution [1]. It has been generally acknowledged that *F. hepatica* was introduced into the Caribbean region in general and Cuba in particular by the infected livestock of Spanish conquistadors between 1500 and 1865 [2–4]. However, the first autochthonous cases of human fascioliasis were not reported in Cuba until 1931 [5]. Fascioliasis was therefore considered to be a public health problem relatively early in the history of Cuba [6]. Although data on human fascioliasis remained rare in the very similar neighbouring island, Hispaniola (Haiti, Dominican Republic) or even nonexistent as in Haiti, animal fascioliasis was well documented in this country [7, 8]. A relatively recent case of fascioliasis was reported in a 26-year-old woman native of Dominican Republic, and living in USA [9], demonstrating the existence of human fascioliasis in Hispaniola. However, to the best of our knowledge, no cases of human fascioliasis have been reported in Haiti, although zoonotic infection of man is known to occur in Cuba, the Dominican Republic and Puerto Rico. Moreover, in Haiti, *F. hepatica* is present in animals and watercress (*Nasturtium officinale*), commonly consumed by the human population. The present study was therefore designed to screen for possible cases of asymptomatic human *F. hepatica* infection in Haiti. A retrospective, cross-sectional, serological survey, testing sera provided by workers, was conducted in Port-au-Prince.

## 2. Material and Methods

Two hundred and sixteen serum samples, provided by workers (18–35 years old) seen in the context of periodic occupational medicine visits, were retrospectively tested (after storage by freezing at −20°C) using a Western blotting test (LDBIO Diagnostics, Lyon, France). The enzyme-linked immunoelectrotransfer blot (EITB) techniques with *Fasciola hepatica* excretory-secretory antigens have been appropriately evaluated for fascioliasis diagnosis in humans [10, 11]. EITB was performed with strips and reagents provided with the kit, according to the manufacturer's instructions. Briefly, the strips were incubated with serum diluted to 1 : 50 in Tris-NaCl sample buffer for 90 min. After a washing step with Tris-NaCl washing buffer, the strips were incubated with an antihuman immunoglobulin G-alkaline phosphatase conjugate for 60 min. After another washing step, the protein fractions recognised by the serum were revealed by the corresponding substrate–chromogenic solution containing nitroblue tetrazolium and 5-bromo-4-chloro-3-indolyl phosphate. The reaction was stopped by washing the strips with distilled water. The strips were dried and glued to paper for reading and storage. A test was considered to be positive when the strip presented 2 or more specific bands including the P27-28 kDa band. Positive and negative controls were tested in each assay.

## 3. Results

Fourteen serum samples were positive on the *F. hepatica* Western blot assay test, each reacting at two to three specific antigenic bands. Under these study conditions, 6.5% of the study population had been in contact with the parasite. As shown in Table 1, the immunodominant bands most frequently recognised by Western blot were 27-28 kDa (100%) and 42 kDa (64%).

## 4. Discussion

*F. hepatica* is a frequently neglected parasitic trematode infecting almost 17 million people worldwide [12, 13]. Recent observations indicate that fascioliasis is gradually expanding and constitutes a serious threat to human and animal health [14]. Indeed, this disease has a great potential for expansion due to the considerable colonisation capacities of its causal agents and vector species [1]. For example, fascioliasis has been detected at very high altitudes (3500–4200 meters) in various Andean regions. The highest prevalence and egg outputs in humans have been observed in these very high altitude zones of Bolivia and Peru [15, 16]. This means that not only snails and parasites are able to successfully colonise the extreme conditions of very high altitude, but also that they have been able to develop various strategies resulting in higher parasite transmission rates. However, the extent of this public health threat is not well known due to poor reporting of cases of fascioliasis worldwide. According to a study conducted by the Pan American Health Organization, fascioliasis is known to cause major losses to the meat industries in Cuba, the Dominican Republic, Haiti, and Jamaica. The extent of losses can be partially quantified by examining the prevalence of bovine liver condemnation, an indicator of morbidity produced by liver-fluke infestation. In the 1960s, liver condemnation rates ranged from 11 to 33% in Cuba [17], 10 to 23% in Saint Lucia, 0 to 3% in Puerto Rico [18], and 60% of all livers were condemned in one Haitian slaughterhouse [19]. In addition to its veterinary and economic impact, potential transmission of *F. hepatica* to humans must not be neglected. Surprisingly, zoonotic infection of man appears to be truly absent from Haiti, as, to our knowledge, there have been no reported cases of human fascioliasis, despite the fact that environmental conditions and eating habits are in favour of human infection. As in other Caribbean countries, animal fascioliasis has been well documented in Haiti [7, 8]. Watercress, which is known to be a potential source of human *F. hepatica* infection, is grown extensively and eaten with nearly every meal in Haiti. It is called “poor man's lettuce” because it is affordable for the majority of the population. Furthermore, sewage systems are rare, and outdoor defecation is common, facilitating human participation in transmission via egg shedding. Animals also usually roam freely. A large distribution of *Lymnaea cubensis* has been reported throughout the country [20]. The absence of case reports of human fascioliasis therefore cannot indicate the absence of this disease in Haiti. Based on variables such as poverty and physician/patient ratio, human fascioliasis should logically be prevalent in Haiti, as supported by the available data, with up to 23% of cattle infected in Haiti [7, 8]. As enzyme-linked immunoelectrotransfer blot is recognised to be a highly sensitive and specific method for the immunodiagnosis of parasite infections, this test was used in the present serological survey. Tests were performed by the same experienced staff to reduce the risk of handling and reading errors. The frequency of antibodies in serum samples of the study population was 6.5% (14/216). However, due to the study design, this result may not indicate the true endemic level of human fascioliasis in Haiti, as fascioliasis is a water-linked disease characterized by a patchy geographical distribution. Haiti, although a relatively small country, has a very heterogeneous geography, ranging from lowlands to highlands, plains to mountains and valleys, and urban and periurban areas to rural areas. This heterogeneous geography would therefore account for the heterogeneous distribution of fascioliasis in Haiti. On the basis of these results, Haiti could be wrongly considered to be a mesoendemic country on the list established by WHO and other agencies. In other words, the positive serology rate of 6.5% should not be applied to the entire country but may correspond to hyperendemic foci diluted in the total of 216 samples. All infected subjects of this study population may be derived from only one or two villages, as geographical data were not available. However, further studies including human stool examination surveys are needed to provide public health authorities with data on the incidence and epidemiology of human fascioliasis in this country. Recent malacological investigations are also needed to identify other potential vectors among snails present in Haiti and to initiate control measures. 

This survey does not claim to provide reliable epidemiological data. However, for the first time, this study provides data on human fascioliasis in Haiti. It is a preliminary report that opens the way for further studies designed to obtain reliable epidemiological data on human fascioliasis in Haiti. Correlation of antibody levels with other features of the disease, such as ova counts in stools, may help to clarify whether asymptomatic infection is due to mild primary infection or to other immune-related factors. In Haiti, these results reinforce the urgent need for control and prevention measures by local public health services. Updated information on diagnosis and management of fascioliasis is required, especially for clinicians who are unfamiliar with the multiple clinical presentations of this disease.

## Figures and Tables

**Table 1:** Frequencies of *F. hepatica* various antigenic fractions reacting with serum from fourteen asymptomatic adults in Port-au-Prince, Haiti.

Sample number	Recognising the following specific bands (kDa)			
	P8-9	P27-28	P42	P60
(1)		+	+	
(2)		+		+
(3)	+^a^	+		
(4)	+	+	+	
(5)		+	+	
(6)		+	+	
(7)		+	+	
(8)		+	+	
(9)		+		+
(10)		+	+	
(11)	+	+		
(12)		+	+	
(13)		+	+	+
(14)	+	+		+

^a^+, Presence of the band.
